# Retinopathy of prematurity protection conferred by uteroplacental insufficiency through erythropoietin signaling in an experimental Murine Model

**DOI:** 10.1038/s41390-023-02568-4

**Published:** 2023-04-04

**Authors:** Camille Fung, Thaonhi Cung, Caroline Nelson, Haibo Wang, Colin Bretz, Aniket Ramshekar, Ashley Brown, Gregory J. Stoddard, M. Elizabeth Hartnett

**Affiliations:** 1 Division of Neonatology, Department of Pediatrics, University of Utah, Salt Lake City, UT USA; 2 Moran Eye Center, University of Utah, Salt Lake City, UT USA; 3 Department of Internal Medicine, University of Utah, Salt Lake City, UT USA; 4 Byers Eye Institute at Stanford University, 2452 Watson Court, Palo Alto, CA 94303 USA

## Abstract

**Background:**

Recent clinical studies suggest that preeclampsia, characterized by uteroplacental insufficiency (UPI) and infant intrauterine growth restriction (IUGR), may be protective against retinopathy of prematurity (ROP) in preterm infants. Experimental models of UPI/IUGR have found an association of erythropoietin (EPO) with less severe oxygen-induced retinopathy (OIR); however, it is unclear if EPO/EPO receptor (EPOR) signaling was involved. We hypothesized that maternal UPI and resultant infant IUGR would protect against features of ROP through EPO/EPOR signaling.

**Methods:**

We compared transgenic mice with hypoactive EPOR signaling (hWtEPOR) to littermate wild-type mice (mWtEpoR) in a novel combined model of IUGR and ROP. Thromboxane A_2_ (TXA_2_) was infused into pregnant C57Bl/6J dams to produce UPI/IUGR; postnatal pups and their foster dams were subjected to a murine OIR model.

**Results:**

Following hyperoxia, hematocrits were similar between littermate wild-type (mWtEpoR) TXA2/OIR and vehicle/OIR pups. mWtEpoR TXA_2_/OIR had increased serum EPO, retinal EPO and VEGF, and decreased avascular retinal area (AVA) compared to vehicle/OIR pups. In comparison to the mWtEpoR TXA_2_/OIR pups, AVA was not reduced in hWtEPOR TXA_2_/OIR pups.

**Conclusion:**

Our findings provide biologic evidence that UPI/OIR-induced endogenous EPOR signaling confers protection against hyperoxia-induced vascular damage that may be related to pathophysiology in ROP.

**Impact:**

- Maternal preeclampsia and infant growth restriction confer retinovascular protection against high oxygen-induced damage through endogenous erythropoietin signaling.

## Introduction

Hypertensive disease of pregnancy (HDP) includes a spectrum of conditions, ranging from gestational hypertension, preeclampsia, HELLP (Hemolysis, Elevated Liver enzymes and Low Platelets) syndrome, and eclampsia. HDP increases the risk for preterm birth^1^ and can lead to poor maternal-fetal nutrient exchange, uteroplacental insufficiency (UPI), and intrauterine growth restriction (IUGR) of the fetus.^2^ A question has arisen as to whether preeclampsia, which lies in the spectrum of HDP, increases the risk of comorbidities of prematurity, such retinopathy of prematurity (ROP).^3–5^ Current medical literature regarding the role of preeclampsia in the risk of ROP is conflicting. Some publications reported a protective effect,^6,7^ whereas others found no association^7^ or increased risk of ROP.^8,9^ A bias exists, because preeclampsia is a risk for premature birth, and ROP occurs only in premature infants. In order to gain insight, investigators performed two analyses from the same group of infants and mothers over a 20-year period in the Intermountain Health Care database.^10^ When a general analysis was performed that included mothers with or without preeclampsia and infants born with or without prematurity, preeclampsia was found to increase the risk for infant ROP. However, after restricting analyses to a sub-cohort of premature, very low-birth weight infants, preeclampsia appeared to be protective. Based on these findings, we were interested to explore whether there were biologic effects from preeclampsia and premature birth that were protective against features that increase risk of ROP.

Experimental models of maternal UPI and infant IUGR have studied retinopathy risk after oxygen-induced retinopathy (OIR). In one study, rat dams were fed a diet low in protein (9% casein) during the final week of gestation.^11^ Pups born to these dams had greater avascular retinal area (AVA) following exposure to high oxygen compared to pups born to dams fed a normal protein (18% casein) diet. These findings suggested that IUGR during exposure to oxygen stresses increased the potential for greater retinopathy in pups by increasing avascular retinal area, thereby aligning with phase I of the hypothesis describing ROP.^5^ The initial increase in avascular retinal area is believed to be a stimulus for later pathologic intravitreal neovascularization in phase II ROP^5^ and may also reduce visual field and vision by compromising vascular support to the retina. However, another study created maternal UPI in rat dams by bilateral partial ligation of the uterine arteries and found reduced avascular retinal area in pups exposed to oxygen-induced retinopathy (OIR),^12^ suggesting a protective effect. Furthermore, the reduced avascular retinal area was associated with increased serum and retinal erythropoietin (EPO) levels compared to pups of dams with sham-surgery. Finding increased EPO raised the question if OIR in IUGR pups induced compensatory mechanisms that were vasoprotective and involved EPO. EPO is increased in the serum of low-birth-weight preterm infants and infants with ROP in non-preeclamptic pregnancies.^13,14^ However, the association of EPO does not imply causation. Or if EPO is expressed, it is unknown if the amount is sufficient to activate signaling in all tissues, particularly the tissue in need. Intriguingly, plasma and placental EPO concentrations were greater in preeclamptic mothers than in control mothers.^15^ In addition, studies in the setting of normal maternal conditions without HDP found that exogenous EPO delivered to pups reduced avascular retina during hyperoxia in mouse OIR.^16^ Therefore the impetus of this current study is to understand whether preeclampsia that leads to maternal UPI and infant IUGR modulate the risk of ROP development via EPO. Also, because none of these studies addressed EPO signaling through its cognate receptor, EPOR, we will investigate this pathway directly via a transgenic mouse showing decreased EPO signaling.

**Fig. 1:**
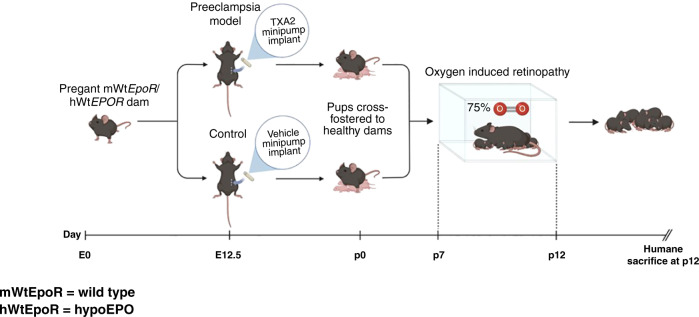
TXA_2_/OIR schematic of the full model including mWtEpoR and hWtEPOR genotypes. TXA_2_ exposure in dams led to UPI and IUGR in pups. Pups and cross foster dams were then placed into hyperoxia (75% oxygen).

Studying the EPO/EPOR signaling pathway is challenging because the antibodies indicating presence or activation of EPOR yield unreliable results, as antibodies for immunohistochemical staining or protein analysis by Western blots have poor specificity.^17,18^ Attempts to create homozygous EPO or EPOR knockout mouse models result in nonviable embryos, further complicating the task of examining EPO-conferred protection in early life.^19^ Therefore, we used transgenic mice in which the murine *EpoR* gene was replaced with the human *EPOR* gene, resulting in hypoactive signaling in mice from a defective transmembrane domain and not ligand-receptor binding.^20^ We designate mice with this transgene as hWtEPOR and littermate control wild-type mice as mWtEpoR. We tested the hypothesis that activation of EPO/EPOR signaling in the condition of maternal HDP, a cause of UPI and infant IUGR, was vasoprotective under high oxygen by studying homozygous hWtEPOR and littermate control mWtEpoR pups in a combined maternal UPI-induced pup IUGR and OIR model (Fig. 1).

## Methods

### Animals

Animal use and procedures were approved by the Institutional Animal Care and Use Committee and Institutional Biosafety Committee of the University of Utah (Salt Lake City, UT) prior to all experiments. Additionally, animal procedures adhered to the University of Utah Guide for the Care and Use of Laboratory Animals and the Association for Research in Vision and Ophthalmology Statement for the Use of Animals in Ophthalmic and Vision Research.

C57Bl/6J mice heterozygous for the mouse wild-type *EpoR* gene (mWtEpoR) and the hWt*EPOR* gene were bred to produce homozygous hWt*EPOR/* hWt*EPOR* (denoted as hWtEPOR) and littermate homozygous murine wild-type *EpoR* (denoted as mWtEpoR) litters. (The hWtEPOR mice were produced and kindly provided by Vladimir Divoky.^20^) In this study, male and female mWtEpoR or hWtEPOR mice were used for experimental analyses. Heterozygotes were not used in analyses. Animals were maintained on 12 h light/dark cycles with *ad libitum* access to food and water. Animal were maintained on a C57/Bl6J background and genotypes were routinely verified by Transnetyx using qPCR.

### IUGR (TXA_2_ Model)

To model HDP and UPI with resultant IUGR in offspring, we infused a thromboxane A_2_-analog (TXA_2_, U-46619) instilled into micro-osmotic pumps in the last week of the mouse gestation as previously described.^21^ On embryonic day 12.5, heterozygous (hWt*EPOR/* mWt*EpoR)* dams were anesthetized by intraperitoneal injection of ketamine (40 μg/g) and xylazine (8 μg/g). Micro-osmotic pumps (model 1007D, 0.5 μl/h, Durect Corporation, Cupertino, CA) containing either vehicle (0.5% ethanol) or 2000 ng/h of U-46619 (TXA_2_ analog, catalog no. 16450, Cayman Chemical, Ann Arbor, MI) were then placed into the retroperitoneum through a 1 cm incision. Dams with TXA_2_ infusion developed hypertension within 24 h of implantation. Implants remained for the remainder of gestation, which is ~20 days in C57Bl/6J mice. Upon birth, litters from implanted heterozygous dams were cross-fostered to non-implanted dams to reduce potential confounding effects from surgery. During our model inception,^21^ we showed that fetal 11-dehydrothromoboxane B_2_ level, a stable TXA_2_ metabolite, was no different in pups born to dams receiving sham or U-46619 infusion, therefore the analog did not cross the placenta to affect the pups directly. As such, we do not believe that a direct interaction between TXA_2_ and EPO would occur in this postnatal model of IUGR and OIR.

### Murine oxygen-induced retinopathy (OIR)

To examine the effects of IUGR in a reproducible model, the murine OIR model^22^ was selected for experimental use. On postnatal (p) day 7, pups were placed into an OxyCycler (Biospherix, Parish, NY) to maintain at 75% oxygen exposure for five days. On p12, pups were returned to room air. The p12 time point represents the phase 1 of the hypothesis of ROP, in which there is avascular retina.^5^ Homozygous hWt*EPOR* (hWtEPOR) and littermate *EpoR* (mWtEpoR) pups were sacrificed on p12 for analyses. Genotypes were treated the same way and pups were euthanized right after being taken from hyperoxia.

### Retinal dissection, staining, and vascular growth measurements

Upon sacrifice, enucleated globes were placed into 4% paraformaldehyde for one hour. A corneal incision was made to promote permeation of the fixative. Following the incubation, intact retinas were removed and cleared of hyaloid vessels and excess vitreous. Four relief incisions were made radially, and retinas were incubated in 488 conjugated *Griffonia* Simplicifolia (Bandeiraea) isolectin–GS-IB4 overnight, as previously described.^23^ Stained retinas were then flat mounted on slides, and the retinal vasculature was visualized with an inverted fluorescence microscope at 20x magnification (Keyence, Illinois). Complete images were captured using Keyence scan-slide stitching software (Molecular Devices, Inc., San Jose, CA). Measurement of avascular retinal and total retinal areas were carried out by two masked observers using ImageJ (NIH, Bethesda, MD) to develop a percent of avascular area/total retinal area (AVA). Adjudication was used to resolve disagreements in measured outcomes between masked observers.

### Hematocrit

Blood samples were collected in duplicate upon sacrifice at p12 and placed into microhematocrit capillary tubes treated with heparin (Avantor, Radnor, PA). Samples were centrifuged five minutes and analyzed with a microhematocrit reader; duplicate measurements were averaged for experimental evaluation.

### Western blot

Dissected retinas were sonicated in radio immunoprecipitation assay lysis buffer supplemented with 1X protease inhibitor (Millipore Sigma, Burlington, MA), and 1X phosphatase inhibitor (ThermoFisher Scientific, Rockford, IL). Lysed samples were centrifuged at 13,000 rpm for five minutes at 4 °C, the supernatants were collected, and protein concentrations were quantified using a Pierce Bicinchoninic Acid Protein Assay Kit (ThermoFisher Scientific, Rockford, IL). The purified retinal proteins were then mixed in sample buffer and denatured at 95 °C for five minutes prior to being subjected to electrophoresis through 4–12% NuPAGE BisTris Gels (Invitrogen, Carlsbad, CA). Proteins were then transferred to an immobilon-P polyvinylidene difluoride membrane and membranes were incubated in 5% bovine serum albumin (BSA) in 1X Tris-buffered saline (TBS, blocking solution) for one hour at room temperature. Membranes were then incubated overnight at 4 °C in blocking buffer supplemented with the following primary antibodies: rabbit anti-EPO (1:500, Santa Cruz Biotechnology, Santa Cruz, CA) or rabbit anti-vascular endothelial growth factor (VEGF, 1:500, Santa Cruz Biotechnology, Santa Cruz, CA). Following overnight incubation, membranes were washed three times in 0.1% Tween-20 TBS solution (washing buffer). Membranes were subsequently incubated for one hour at room temperature in blocking solution supplemented with the species appropriate secondary antibody, horseradish peroxidase (HRP) conjugated goat anti-rabbit (1:3000, Cell Signaling Technology, Danvers, MA), and HRP conjugated β-actin (1:3000, Santa Cruz Biotechnology, Dallas, TX). Images were captured using the C-DiGit Blot Scanner (LI-COR Biotechnology, Lincoln, NE) and Image Studio software (v5.2, LI-COR Biotechnology, Lincoln, NE). FIJI software was used to perform densitometry analysis, and the results were normalized to β-actin.

### ELISA

Blood samples collected at sacrifice on p12 were maintained at 4 °C and allowed to coagulate for two hours in non-heparin-treated tubes then centrifugated for 25 min at 2000 × *g*. Serum supernatant was assayed for EPO and VEGF using Mouse EPO and VEGF Enzyme Linked Immunosorbent Assay kits (ELISAs; R&D, Minneapolis, MN) following manufacturer’s protocols.

### Statistical Analysis

All sample size estimates were determined with assistance from a biostatistician (GJS). At least 3 litters were used for each question to account for biologic variability and after accounting for the design effect of lack of independence from data clustering, 5 pups/group were required for 80% power with a two-sided alpha 0.05 comparison for the primary outcome of AVA. Secondary analyses included body weight and western blots.

All statistical analyses were performed in Stata-17 (StataCorp LLC, College Station, TX). For the in vivo experiments, the data were analyzed with a mixed effects linear regression model with eyes nested with animal (for eye level variables), animals nested with litters, and litters nested within individual experimental settings, using AVA, western blot densitometries, quantitative ELISA data, or weight as the continuous outcome variables. Predictor variables were implant group limited to a specific genotype for some models, and genotype-implant combinations for other models. Results are presented as mean ± standard error (SE), and a *p* value < 0.05 was considered statistically significant.

## Results

### Increased endogenous EPO expression in the IUGR model after high oxygen

**Fig. 2:**
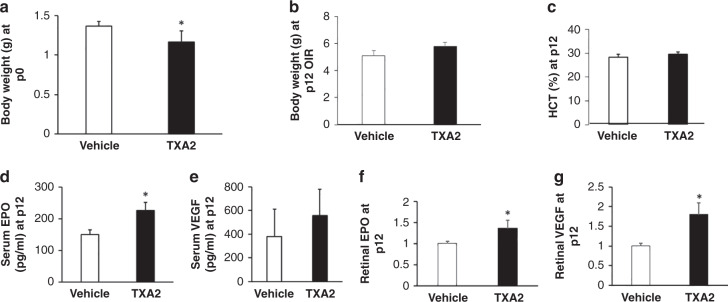
TXA_2_/OIR model on pup parameters. **a** Birth weights of mWtEpoR pups born to dams infused with vehicle or TXA_2_ (p0 weight: vehicle (*n* = 3 pups), TXA_2_ (*n* = 5 pups)). **b** Body weights of mWtEpoR pups in OIR at p12 born to dams infused with vehicle or TXA_2_ (p12 weight: vehicle (*n* = 10 pups), TXA_2_ (*n* = 7 pups)). **c** Hematocrit of mWtEpoR pups in OIR born to dams infused with vehicle or TXA_2_ (p12 hematocrit: vehicle (*n* = 13 pups), TXA_2_ (*n* = 11 pups)). **d** Serum EPO and (**e**) VEGF of mWtEpoR pups in OIR born to dams infused with vehicle or TXA_2_ (p12 serum EPO: vehicle (*n* = 5 pups), TXA_2_ (*n* = 6 pups); p12 serum VEGF: vehicle (*n* = 6 pups), TXA_2_ (*n* = 6 pups)), (**f**) Retinal EPO or (**g**) VEGF of mWtEpoR pups in OIR born to dams infused with vehicle or TXA_2_ (p12 retinal EPO: vehicle (*n* = 8 pups), TXA_2_ (*n* = 8 pups), p12 retinal VEGF: vehicle (*n* = 5 pups), TXA_2_ (*n* = 5 pups). Results are represented as mean ± SEM, *p < 0.05 compared to vehicle mWtEpoR.

Compared to the vehicle controls, pups naturally born to dams infused with TXA_2_ had lower birth weights (*p* = 0.035, Fig. 2a) duplicating our previous finding in IUGR.^21^ Following OIR at p12, catch-up growth occurred in pups born to TXA_2_-exposed dams and placed into OIR (TXA2/OIR) to cause weights that were commensurate with those of pups born to dams implanted with vehicle (vehicle/OIR) (Fig. 2a, b). There was no significant difference in hematocrit between pups in the TXA2/OIR group and pups in the vehicle/OIR group at p12 (*p* = 0.387, Fig. 2c). However, serum EPO (*p* = 0.027, Fig. 2d), but not serum VEGF (*p* = 0.417, Fig. 2e), was significantly increased in pups born to TXA2/OIR group compared to vehicle/OIR group at p12. In addition, retinal EPO (*p* = 0.031, Fig. 2f) and retinal VEGF (*p* = 0.013, Fig. 2g) were increased in TXA2/OIR compared to vehicle/OIR at p12. Therefore, the murine TXA_2_/OIR model demonstrated catch-up growth and increased serum EPO aligning with a previous rat IUGR/OIR model.^12^ Also, hematocrit, which clinically affects ROP,^24^ was no different between groups at the p12 time point. These outcomes provided confidence to use the TXA_2_ model at p12 to test our hypothesis.

### TXA_2_/OIR model reduces vascular loss in hyperoxia due to EPOR signaling

**Fig. 3:**
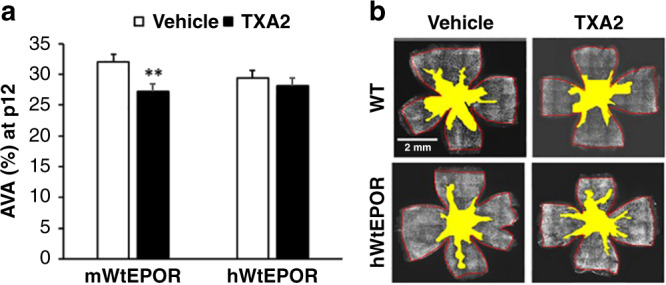
Effects of endogenous EPOR signaling on AVA. **a** Percent avascular/total retinal area (AVA) at p12 of mWtEpoR or hWtEPOR pups in OIR born to dams infused with vehicle or TXA_2_ [mWtEpoR /vehicle (*n* = 13 pups), mWtEpoR/TXA_2_ (*n* = 11 pups), hWtEPOR/vehicle (*n* = 14 pups), hWtEPOR/TXA2 (*n* = 11 pups)], and (**b**) representative retinal flat mount images with filled-in AVA. (*p < 0.05; **p < 0.001).

We then sought to determine if maternal UPI, which increased pup serum and retinal EPO, lent protection to pup retinal capillaries following high oxygen. To assess this postulate, we measured AVA at p12 in retinal flat mounts from TXA_2_/OIR and vehicle/OIR pups. Compared to 30.8% AVA in the vehicle/OIR group, AVA was significantly decreased to 27.9% in the TXA_2_/OIR group at p12 (Fig. 3a, b), supporting the notion that maternal UPI was associated with pup retinal vasoprotection during high oxygen. To investigate the role of endogenously-induced EPO signaling through its receptor, EPOR, on vasoprotection, we compared pups born to TXA_2_-implanted or vehicle-implanted dams in the hypoactive EPOR-signaling transgenic mouse model.^20^ With hyperoxia alone, there was a pattern towards reduced AVA in the vehicle exposed hWtEPOR mice compared to the vehicle exposed wild-type mice but it did not reach statistical significance. In contrast to the reduced AVA found from TXA_2_/OIR in mWtEPOR, there was no significant difference in AVA between the vehicle/OIR and TXA_2_/OIR hWtEPOR pups (Fig. 3a, b). Taken together, the data support the postulate that endogenously-induced EPOR signaling participates in vascular protection in pup retina following high oxygen.

## Discussion

With the advancement in obstetric and neonatal care, younger gestational age and extremely low-birth weight infants are surviving. Survival may be accompanied by morbidities, such as blindness and neurodevelopmental delays. One common pregnancy complication that often leads to the need for early delivery is HDP whereby diminished uteroplacental blood flow compromises fetal growth causing IUGR. Controversy currently exists as to whether HDP spectrum of diseases is protective or detrimental to the development of retinal vessels in these highly vulnerable infants after exposure to ex utero hyperoxia. Furthermore, the molecular mechanisms underlying such potential protection remain elusive.

In this study, we found that pups born to dams exposed to TXA_2_ had increased serum and retinal EPO with associated reduction in AVA following hyperoxia. In alignment with the previous findings in the mouse OIR model, retinal VEGF was also increased.^22^ We previously observed similar increased EPO and reduced AVA in a rat UPI/OIR model.^12^ In face of hyperoxia, downregulation of EPO, which can be induced by hypoxia-inducible factors, might have occurred. All the dams in hyperoxia were heterozygous, so it is not certain that EPO came from a maternal source. However, in the rat UPI/OIR model, because EPO does not cross the placenta, the source of EPO was from the pup and believed to be a tissue protective response.^12^ We postulated that increased EPO binding to its EPOR would then activate EPO signaling to reduce AVA in mWtEpoR/OIR pups born to dams exposed to TXA_2_. This observation suggests that EPO signaling is involved in the vasoprotection following hyperoxia. To test this postulate, we used a hypoactive EPOR-signaling mouse model in OIR (hWtEPOR/OIR) and found that pups born to dams implanted with either vehicle or TXA_2_ had similar AVA. These findings contrast with mWtEpoR/OIR mice born to dams implanted with TXA_2_ that had increased EPO and reduced AVA following hyperoxia compared to vehicle-infused mWtEpoR/OIR mice. Hypoactive EPOR-signaling in hWtEPOR mice is due to the human transmembrane EPOR domains having a lower affinity towards the formation of homodimers, reducing overall erythropoietic activity when expressed on the murine cell membrane rather than due to receptor/ligand binding.^25^ This transgenic model thus provides a reliable means to assess EPOR-signaling in vivo since antibodies used to assess EPOR or its activation do not have the necessary specificity for analysis in immunohistochemistry^17,18^. We recognize that this transgenic model is not specific to the retina or the vasculature, and future studies assessing the role of EPOR signaling in endothelial or other cell types in the retina will be important.

The hWtEPOR mice are known to have high circulating EPO as a compensatory effect of the hWtEPOR genotype.^20^ Serum EPO level may trigger other signaling mechanisms and affect outcomes through its hormonal effects or through local tissue effects, such as the EPOR/β common receptor (βCR) or VEGFR2-EPOR pathways.^26,27^ The βCR has been implicated in tissue protective effects. The tissue protective receptor has subunits that associate following tissue injury and hypoxia composed of the βCR, EPOR, and in some tissue types, VEGFR.^28^ Under hypoxic conditions, VEGFR2 forms complexes with βCR-EPOR to support tissue health by inducing the production of nitric oxide.^28^ These mechanisms might additionally explain potential protective interactions between increased retinal VEGF in TXA2/OIR mice and EPOR signaling.^28,29^ In addition, these alternative mechanisms may explain the apparent pattern of reduced AVA in vehicle exposed hWtEPOR mice compared to the vehicle exposed murine wild-type mice, both in OIR. In contrast to other experimental or clinical studies of exogenous EPO^30–33^, the current study addressed endogenously-induced EPOR signaling rather than exogenous EPO delivery and, therefore, may not be comparable. Longitudinal clinical studies, including the recent PENUT trial, examined the efficacy of high dose exogenous EPO in preterm infants.^34,35^ These studies did not observe a significant difference in the neurodevelopmental outcomes or rates of ROP in preterm infants administered exogenous EPO compared to placebo. Results were limited by available methods for analysis of neurocognition in two- and five-year old children and relatively short duration of the studies.^34,36^ The cohort of patients with documented ROP in the PENUT study was small, further complicating the interpretation of the results. Follow up on the participants of these studies through anatomic maturation will be needed to assess neurocognitive studies in older children as well as in their retinal structure and function. In addition, there may be other yet unclear mechanisms how endogenous EPOR signaling may have benefits not seen with exogenous EPO or that triggering of endogenous EPOR signaling may interact with exogenous EPO delivery in ways that are unexpected. Also, lower dose EPO or derivatives of EPO, such as darbepoetin, are routinely used to prevent anemia of prematurity in infants and have been reported to have benefit in some clinical studies.^37^ Therefore, studying EPO or derivatives and their signaling effects on features involved in ROP pathophysiology is highly important and clinically relevant.^33^

We recognize certain limitations exist in our study. Human ROP is a disease of prematurity, whereas the mouse develops its retinal vasculature postnatally and does not experience the stresses of prematurity.^5^ It is this predictability in vascular plexus development in normoxia that makes the study robust in its design when a secondary insult such as hyperoxia is imposed. We also understand that we have not dissected the interactions between EPO and VEGF in this model given that VEGF is a major driving force behind ROP development. Studies are ongoing to understand the interplay between EPO and VEGF.

In conclusion, our study is the first to combine two well-established mouse models of maternal HDP and infant IUGR with OIR to evaluate the effects on high oxygen-induced vascular damage. Our current observations support the hypothesis that HDP confers retinal vasoprotection to offspring during high oxygen in part via endogenous EPOR signaling. Future studies are indicated to assess if UPI confers long term protection.

## Data Availability

Data will be available upon request and after publication.
